# Neglected Tropical Diseases in the Anthropocene: The Cases of Zika, Ebola, and Other Infections

**DOI:** 10.1371/journal.pntd.0004648

**Published:** 2016-04-08

**Authors:** Peter J. Hotez

**Affiliations:** 1 Departments of Pediatrics and Molecular Virology and Microbiology, National School of Tropical Medicine, Baylor College of Medicine, Houston, Texas, United States of America; 2 Sabin Vaccine Institute and Texas Children’s Hospital Center for Vaccine Development, Houston, Texas, United States of America; 3 James A. Baker III Institute for Public Policy, Rice University, Houston, Texas, United States of America; 4 Department of Biology, Baylor University, Waco, Texas, United States of America; University of Texas Medical Branch, UNITED STATES

While we advance through a geological epoch that increasingly reflects human intervention on a massive scale, we might expect to see the continued expansion of epidemic neglected tropical diseases, as we have recently seen for Zika and Ebola virus infections.

Emerging evidence indicates that the Holocene, our most recent geological epoch that began at the end of the last ice age almost 12,000 years ago, has undergone some fundamental changes because of human activity. Since the origins of agriculture and deforestation and later accelerating with the industrial revolution, followed by rapid 20^th^ century population growth extending into the nuclear age, our planet has undergone a fundamental and seemingly irreversible geological shift [1]. According to many (but not all) prominent Earth scientists, humans have profoundly altered the planet, thereby ushering in a new and so-called Anthropocene epoch (Fig 1).

**Fig 1:**
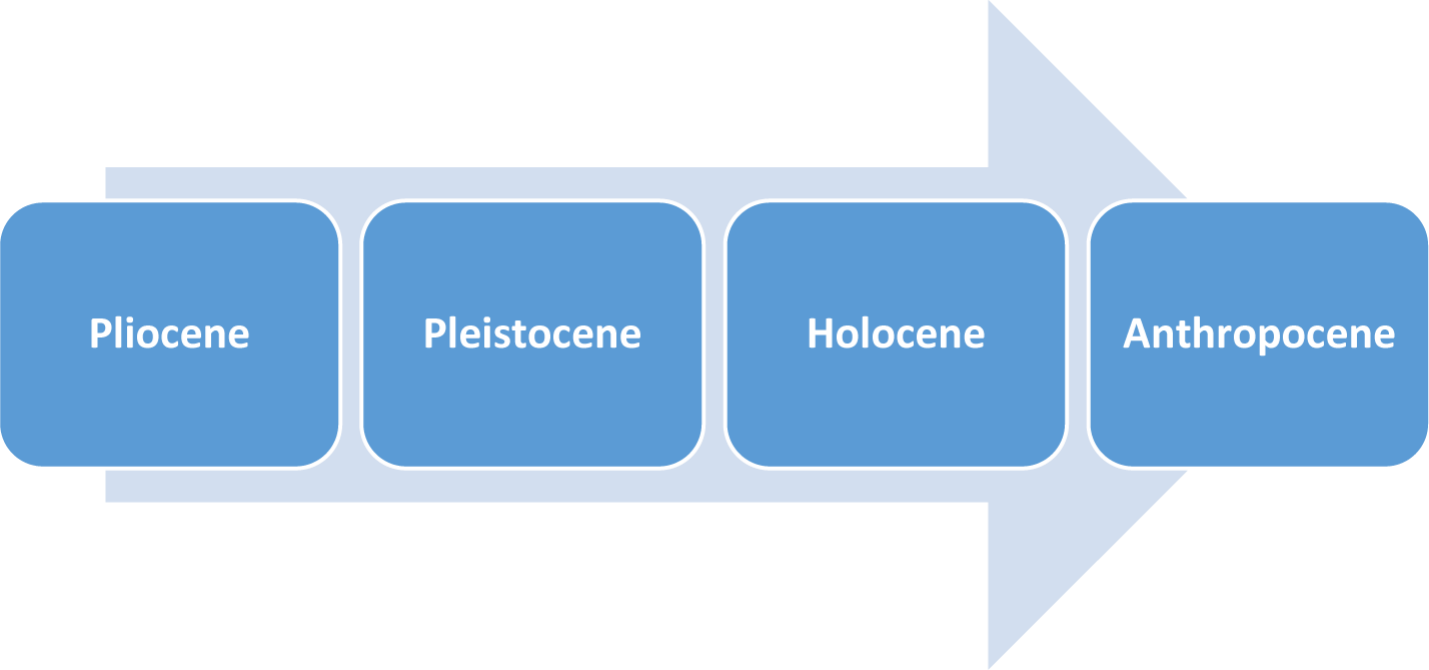
Geological epochs over the last 5 million years.

In a January 2016 article in *Science*, Colin Waters from the British Geological Survey and his colleagues provide important geochemical evidence to support designating the end of the Holocene as the Anthropocene [1]. It includes data showing increasing lead levels after World War II, altered soil nitrogen and phosphorous levels because of increased fertilizer use, and the appearance of newly created radionuclides, beginning with the atomic bomb tests in the New Mexico desert at Los Alamos [1]. Alongside these human-induced geochemical signatures are elevated carbon dioxide and methane levels and sharp increases in average global temperatures [1].

Levels of concrete and plastic have also dramatically increased in recent years, while in parallel, there has been massive loss of animal and plant species [2]. Species extinctions have reached unprecedented levels [1,3].

In this late Anthropocene epoch, we have seen significant increases in the incidence or prevalence rates of several neglected tropical diseases (NTDs), due partly or mostly to human-induced changes to our planet. This is especially true for NTDs transmitted by invertebrate vectors, including mosquitoes, kissing bugs, and snails, as well as highly lethal zoonotic virus infections from bats and other mammals. For example, in the Americas, dengue fever reemerged in the 1980s, while chikungunya and Zika virus infections have aggressively spread across the Latin American and Caribbean region. Venezuela in particular has seen dramatic increases in malaria and most of its neglected tropical diseases (NTDs), including Chagas disease, schistosomiasis, and Zika virus infection, for which unprecedented urban foci are also occurring [4]. Across the Atlantic Ocean, Southern Europe has of late seen the emergence or reemergence of malaria in Greece, West Nile virus infection and chikungunya in Italy and Spain, dengue in Portugal, and schistosomiasis on the French island of Corsica [5]. The Middle East and North Africa (MENA) region is now considered one of the worst-affected global hotspots for NTDs and other emerging infections such as leishmaniasis, schistosomiasis, and MERS coronavirus infection; measles and polio have also returned [6]. Ebola caused thousands of deaths and overwhelmed the health systems of Guinea, Liberia, and Sierra Leone in West Africa in 2014–2015 [7], while East Africa and the Sahel are considered among the most important regions for kala-azar and multiple other NTDs [8]. Schistosomiasis continues to increase throughout Africa, where it is now a major cofactor in its AIDS epidemic [9]. Southeast Asia has seen the rise of Nipah and Hendra virus from bats, in addition to drug resistant malaria, enterovirus 71, melioidosis, and foodborne trematodiases transmitted by snails [10].

Several human activities that characterize the Anthropocene account for the increases in NTDs. It is instructive to see how some of these factors illustrated in Fig 2 helped to facilitate the emergence of two of the most devastating NTDs in 2014 and 2015—Ebola and Zika virus infections, respectively, as well as other high-disease-burden NTDs such as the cutaneous and visceral forms of leishmaniasis and schistosomiasis.

**Fig 2:**
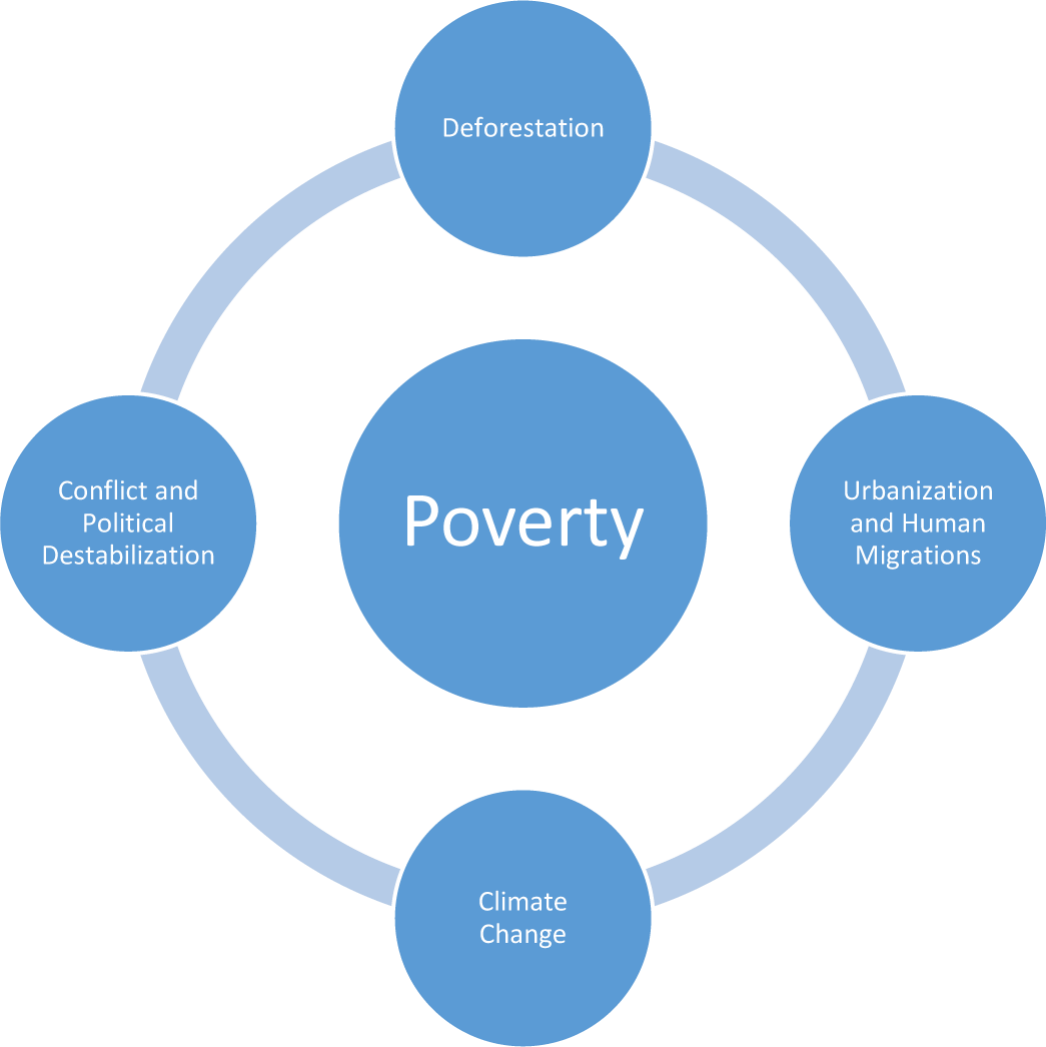
The major forces arising out of the Anthropocene now promoting the emergence of catastrophic neglected tropical diseases (NTDs).

## Poverty and Blue Marble Health

As has been said very frequently in our editorials, poverty is front and center. NTDs are most common in the setting of poverty [11,12], while simultaneously helping to perpetuate poverty through their long-standing negative effects on maternal and child health and human productivity and labor [13,14]. Ebola has so far emerged almost exclusively in impoverished nations such as the Democratic Republic of Congo or Guinea, Liberia, and Sierra Leone, while Zika is disproportionately affecting impoverished areas such as Brazil’s poorest northeastern provinces. In the case of Zika or other vector-borne NTDs such as leishmaniasis and Chagas disease, poverty equates to poor quality housing, in addition to uncollected garbage and standing water in poor neighborhoods that allow certain insects to breed nearby. For these reasons, we might expect poor countries such as Haiti or Jamaica to suffer greatly from the advance of Zika in the Caribbean region.

Yet another feature of Zika, leishmaniasis, Chagas disease, and other NTDs are their propensity to strike the poorest people who live in the wealthier group of 20 countries, such as Brazil or Mexico. The concept of “blue marble health” has been invoked to describe the surprising disease burden of NTDs among the poor living in these countries [15]. Today, most of the world’s NTDs paradoxically occur in the world’s largest economies, but mostly among the disenfranchised poor in those nations [15].

## Political Destabilization, Conflict, and Post-conflict

Next to poverty, these forces may account for the largest risk factor for NTDs [6,16,17]. The long-standing atrocities and civil and international conflicts decimated the health systems of Guinea, Liberia, and Sierra Leone, thereby allowing Ebola and Lassa fever to flourish [7,17], while these same forces facilitated the rapid spread and lethality of human African trypanosomiasis and kala-azar in Africa [8,18]. Conflict and post-conflict settings are central to the massive epidemic of cutaneous leishmaniasis in the Middle East [6]. While Zika has so far not been linked to these factors, the political destabilization in Venezuela could become a contributory factor.

## Deforestation

In Asia, deforestation may have increased human and bat contact to promote Nipah and Hendra viruses and SARS [10]. Deforestation is also an important factor promoting the expansion of vector borne NTDs, including leishmaniasis [19]. Deforestation has been noted to have some possible links with the emergence of Ebola [20]. For instance, the Guinea forest region where Ebola emerged in 2014 has been severely and adversely affected by clear-cut logging [7].

## Dams

Bodies of fresh water arising from large-scale hydroelectric projects can help aquatic snails to proliferate, including those that transmit schistosomiasis in Africa and foodborne trematodiases in Asia. Dams and newly formed reservoirs of fresh water also create mosquito breeding sites for arbovirus infections and malaria and also facilitate waterborne intestinal infections [21]. In China, on the other hand, the Three Gorges Dam on the Yangtze River has assisted flood control and so far has not been shown to promote the emergence of *Schistosoma japonicum* infection [22].

## Urbanization and Human Migrations

The large-scale movement of human populations into cities can create crowded conditions, which together with destruction of the environment favor arthropod vectors [23], including the *Aedes aegypti* mosquito that transmits Zika, dengue, chikungunya, and yellow fever. Accelerated urbanization that outpaces sanitation and sewage control infrastructures is also a key factor in the endemicity of leptospirosis and enteric NTDs [24,25]. Overall, human migrations of immunologically naïve populations to endemic regions have accounted for rapid spread of infections, as well as the converse—infected populations introducing new diseases. The 2014 Brazilian FIFA World Cup soccer games may have been a factor in bringing Asian populations with Zika into areas where *Aedes aegypti* is found, although this hypothesis has since been dismissed [26]. However, the annual Hajj, the Muslim pilgrimage to Mecca that brings millions of people to Saudi Arabia, has also been postulated as having helped introduce dengue [27,28], as it could Zika virus infection in 2016 or 2017. The Hajj has also had a role in the emergence of meningococcal disease and other acute respiratory infections [29,30].

## Climate Change and El Niño Events

Major climate change events in the Anthropocene include increased temperatures and altered rainfall patterns that expand arthropod vector habitats and ranges. Such factors could be responsible for accelerating the geographic expansions of arbovirus infections [31,32], leishmaniasis [33], and Chagas disease [34], but this concept requires further exploration. In addition, we are just beginning to understand the role of El Niño events in promoting these and other NTDs [35,36]. The fact that Zika virus expanded dramatically in an El Niño year is also of interest, but as yet, there are no proven links.

Many of the factors highlighted above were either manufactured or shaped by human activity. The dramatic expansions in the number of cases of arbovirus infections caused by dengue, chikungunya, and now Zika in recent years, together with a recent explosive Ebola outbreak in 2014–2015, give us pause to evaluate human influence on the biosphere and to recognize that beyond globalization, the Anthropocene could become a dominant theme for spreading NTDs or creating catastrophic human epidemics in the years to come.
